# Comparative Effects of a Cow Hoof Cover^®^ and Bandaging as Dressing Materials for the Treatment of Digital Dermatitis in Dairy Cows

**DOI:** 10.3390/ani16213468

**Published:** 2026-11-03

**Authors:** Mohammed Babatunde Sadiq, Siti Zubaidah Ramanoon, Z. A. Jelan

**Affiliations:** Department of Farm and Exotic Animal Medicine and Surgery, Faculty of Veterinary Medicine, Universiti Putra Malaysia (UPM), Serdang 43400, Selangor, Malaysia

**Keywords:** animal welfare, bandaging, Cow Hoof Cover**^®^**, dairy cattle, digital dermatitis, lameness

## Abstract

Digital dermatitis (DD) contributes to lameness, posing major animal welfare and economic concerns. Corrective trimming, topical antibiotics, and bandaging are widely practised in managing DD. However, the challenges associated with conventional bandaging include water retention and a moist environment, which affect the healing process. This study evaluated the effectiveness of a Cow Hoof Cover^®^ (CHC) for the treatment of DD in dairy cows. A quasi-experimental study was conducted, involving cows affected with active stages of DD (M1 and M2), allocated to CHC (*n* = 29) and conventional bandaging (*n* = 27) groups. The standard treatment entailed a Dutch five-step hoof trimming, wound management, application of topical antibiotic containing oxytetracycline (Engemycin 25 mg/mL), and wrapping of the digit using either the CHC or conventional bandaging. All cows received an intramuscular injection of flunixin meglumine (2.2 mg/kg) for pain relief. Primary outcomes were healing of DD lesions (transition from active stages to M0) and restoration of normal gait (locomotion score of 1). All cows were observed weekly until Week 4 after treatment. While alterations in locomotion score were not significantly different between the groups (*p* = 0.23) during the study period, the CHC group had significantly higher (*p* < 0.01) proportions of cured cows at week 3 (*n* = 6, 20.6%) and week 4 (*n* = 17; 58.6%) compared to the control. None of the CHC-wrapped lesions progressed into the chronic hyperkeratotic or proliferative stage (M4), but such transitions were recorded in four cows in the control group. Overall, the CHC had significantly higher probability of cure (HR = 2.79; 95% CI 1.30–5.97) than the control group. Excluding the treatment group, none of the tested animal characteristics and treatment-related variables influenced the probability of cure. Overall, the CHC offers practical advantages over conventional bandaging; it is quick to apply, durable, and water-resistant, supporting its use for field application and large-scale herd management. However, histological investigation and robust clinical trials are recommended to ascertain whether the CHC promotes a complete cure of active DD lesions.

## Simple Summary

Digital dermatitis (DD) is a common cause of lameness in dairy cows and can negatively affect animal welfare and farm productivity. This study compared a Cow Hoof Cover^®^ (CHC) with conventional bandaging for treating DD in dairy cows. The recovery from lameness and transition of DD lesions from painful to non-painful stages occurred faster in cows treated with CHC by day 21 after the treatment. The CHC is a quick, durable, and water-resistant alternative to conventional bandaging, although further clinical studies are needed to confirm complete healing of DD lesions.

## 1. Introduction

Digital dermatitis, a polymicrobial infection, is a common cause of lameness in dairy cows [1,2], beef cattle [3] and small ruminants [4]. While several bacterial species are present in DD lesions, Treponemes (*Treponema phagedenis, T. pedis*, and *T. vicentii*) are widely identified as the major pathogens [5,6]. Digital dermatitis constitutes a significant concern to animal welfare and productivity, with economic losses resulting from reduced milk yield [6,7], poor reproductive performance [8] and the high cost of maintaining affected cows in the herd [9].

The mean prevalence of DD in dairy production can exceed 30%, which is largely influenced by seasonality and associated with winter housing [10,11,12]. Although clinical lameness may not result from DD lesions, poor control measures often increase the risk of active DD lesions, leading to gait disturbance [13,14]. A well-established, effective control method is the targeted treatment of infected cows, performed hygienically to prevent further spread. This strategy is combined with preventive measures such as environmental hygiene (clean and dry walking surfaces), biocontainment (preventing infection of youngstock), biosecurity (closed herd), foot cleaning and disinfection protocols such as foot bathing [1,15,16].

Treponemes are sensitive to a range of antibiotics, including penicillin, erythromycin and oxytetracycline [17]. A single or repeated application of tetracycline spray is highly effective for DD treatment, as reviewed by Potterton et al. [18]. Despite the availability of various treatment modalities, injectable antibiotic therapy is insufficient for a complete cure, given the hyperkeratotic and necrotic characteristics of most DD lesions [19]. Cleaning, debridement and topical treatment are considered more effective and highly recommended because of lower antimicrobial usage and less risk of resistance selection pressure [1].

Non-antibiotic treatments for DD include the application of Hoof-fit Gel^®^ (Intracare, Veghel, The Netherlands)—a product containing activated copper and zinc chelate. Recent studies revealed the comparative efficacy of this non-antibiotic therapy and other antibiotic treatments [20,21,22]. Salicylic acid is another non-antibiotic medication used for DD treatment [16]. Ketoprofen reduces the pain and lameness associated with DD lesions and other causes of lameness, particularly when combined with oxytetracycline [6].

While topical antibiotic and non-antibiotic treatments are pertinent for DD control, evidence-based data on bandaging remains poorly understood. Only a few clinical trials have considered the effects of bandaging in DD treatment. Cutler et al. [23] evaluated treatment with and without a bandage and reported healing rates at 3–7 days and 8–12 days post-intervention. Bandaging displayed no benefit as no significant difference was detected in the tested outcomes. Moreover, post hoc power calculation revealed that more than twice the sample size is required to ascertain if the observed effect size would be statistically significant. The follow-up period was also relatively short to monitor the transition from active lesion to complete healing.

Klawitter et al. [22] compared activated copper/zinc chelate and chlortetracycline applied with or without a bandage (four-layered dressing: gauze, cotton wool, non-elastic bandage and a layer of beech tar). Bandage led to a significantly higher cure rate (i.e., defined as M0—absence of visible lesion) relative to no bandage, but no significant difference between copper gel and antibiotic treatments. Fiedler et al. [24] investigated the use of polyurethane dressings with and without topical antibiotic therapies. Specifically, the groups comprised a single oxytetracycline application without bandaging, salicylic acid with 5-day bandaging, and a polyurethane bandage applied for 10 days without topical antibiotics. At the end of 36 days of follow-up, cure rates were not significantly different between the groups. However, all treatments were better compared to the negative control at day 10, reflecting that bandaging alone is as important as topical agents. Indications against bandage use include the risk of bandage injuries [24].

Typical bandages become wet within days post-application, particularly in pasture-based systems and confined housing with daily foot bathing. Such wet conditions are associated with poor outcomes such as macerated skin, delayed wound healing and a high risk of new infections. The failure to remove bandages heightens the risk of skin cuts and ischaemic injuries to the distal limb [25]. Hence, recommendations to address these side effects range from no bandage to the usage of light dressings, while others suggest using a thick bandage to prevent moisture ingress. Overall, keeping DD lesions dry and out of slurry remains the ultimate goal.

In this study, we investigated the efficacy of a novel dressing material, Cow Hoof Cover**^®^** (CHC), as a treatment modality for active DD lesions, particularly M1 and M2. The CHC is made up of a water-resistant latex material that is easier to apply and detach. We hypothesised that the combination of topical treatments and CHC would facilitate higher cure rates of active DD lesions compared to conventional bandaging.

## 2. Materials and Methods

### 2.1. Study Design and Ethical Approval

A quasi-experimental study was conducted involving cows affected with DD managed using the CHC material (CHC) and a historical control group managed using the conventional bandaging (Control). This study is part of a larger project focusing on novel treatment modalities for lameness in dairy cows. Approval was obtained from the Institutional Animal Use and Care, Universiti Putra Malaysia (Reference no:AUP-R041/2024).

### 2.2. Farm Selection

Farm selection was based on herd size, proximity to the research institution, and farmers’ willingness to participate in the research. A commercial dairy farm located in Pahang, Malaysia, with a herd size of 2550 cows, was selected for this study. The cows were Australian Sahiwal and Jersey Crossbred. During this study, approximately 1400 cows were in various lactation stages, producing an average milk yield of 18–25 litres/day. A rotary system is used in milking the cows twice daily (7:00 am and 4:30 pm). The cows are confined throughout lactation and kept in free stalls. Each stall is designed with in-place rubber mats, and the surface is enriched with calcium carbonate powder to improve cow comfort.

Functional hoof trimming (i.e., five-step Dutch method) is performed by a well-trained staff, specifically for cows in early (within 120 days in milk; DIM) and late lactation (above 220 DIM). Data on hoof health, including the type and severity of claw lesions, claws affected, and the procedures performed, are collected electronically during hoof trimming. Lame cows are identified by daily locomotion scoring as per routine management. Non-lame cows are subjected to foot bathing (i.e., containing copper sulphate) immediately after milking. The footbath is located at the exit of the rotary milking system and is changed weekly by dissolving 20 g of copper sulphate in 1 litre of water [18]. Meanwhile, lame cows are kept in a rehabilitation pen located near the hoof care unit until recovery. These cows are not exposed to the footbath designed for routine hoof care as a preventive measure for healthy lactating cows.

In terms of farm hygiene, floors and pathways at the milking parlour, resting barn, walkway, and holding yards are cleaned twice daily using motorised scrapers and manual water pumping systems. The cows are fed a total mixed ration composed of Napier grass, soybean cake, fish meal, grain corn, and other supplements, which are adjusted according to the stages of lactation.

### 2.3. Sample Size and Sampling Procedure

The minimum required sample of cows with active DD lesions (M1 and M2) in the CHC and Control groups was 25 each, leading to a total of 50 cows. The recruitment of cows into the CHC group started on 8 November 2024. Cows fulfilling the eligibility criteria were allocated directly to the CHC group, whereas a historical control was randomly selected from the available farm records on cows treated for DD prior to the onset of this clinical trial. Specifically, farm records between 29 November 2023 and 30 May 2024 were checked, and historical controls were randomly selected using computer-generated numbers. This period was selected to adjust for possible environmental influences, such as weather and seasonal variation. We prioritised records closest to the onset of the clinical trial to ensure that the historical controls are similar to the active CHC group. This procedure was performed each time cows were allocated to the CHC group until the required sample size was obtained.

Specific measures were taken to ascertain the validity of the DD cases, particularly to adequately demonstrate that the environmental and management conditions remain unchanged between the historical and prospective periods. Hoof-trimming personnel and expertise, diagnostic procedures and lesion classification criteria, herd management protocols, footbath management, follow-up procedures, record quality and completeness were the same during the selected periods. There were no changes in terms of treatment modalities, except for the introduction of CHC for the new DD cases.

### 2.4. Animals Selection and Enrolment Criteria

Cow selection and enrolment for the treatment group began in September 2024 until February 2025. All the lactating cows in the farm were assessed for locomotion scores (i.e., five-point locomotion scoring developed by Sprecher et al. [26] by trained personnel daily as the cows exited the rotary milking system. Cows with a clinical diagnosis of active DD lesion (M1 or M2 stages) following an observation of the digits while standing in the rotary milking system and those with a locomotion score of either two (mildly lame) or three (moderately lame) were selected for further examination. Comparisons of weekly locomotion scores revealed acceptable intra-observer reliability with a Kappa coefficient of 0.90.

In order to enrol cows that are similar to the historical control, the eligibility criteria were developed to align with the routine lameness and hoof care programme in the study farm. The inclusion criteria entailed lactating primiparous or multiparous cows with a first DD-associated lameness event on any of the limbs, locomotion score of two or above, absence of other foot lesions of either infectious (i.e., heel horn erosion and foot rot) or non-infectious origin (sole ulcers, sole haemorrhage, white line disease, thin sole, toe ulcer, etc.). Cows presenting with an active DD lesion (M1 and M2 stages) were eligible for the study. Meanwhile, animals not conforming to the aforementioned eligibility criteria were excluded. In addition, we did not enrol cows that received parenteral antibiotics or anti-inflammatory and analgesic drugs within the previous 2 weeks, thin cows with BCS < 3.0, or those within 10 DIM. This information was considered in the inclusion criteria based on the available farm data and records on previous DD cases.

The eligible cows were restrained in a hydraulic hoof-trimming chute for hoof examination. Detection of the lame foot entailed a multifaceted approach: locomotion scores during mobility assessment, visual observation for the presence of active DD lesions, and withdrawal reflex upon applying pressure to the claw zones using a hoof tester. The classification and description of DD lesions are presented in Table 1. A veterinarian expert in ruminant medicine and bovine lameness research performed the clinical diagnosis of all the claw lesions. The International Committee for Animal Recording (ICAR) claw health classification was also used as a guide when necessary [27]. Upon fulfilling the inclusion criteria, cows were enrolled into the treatment group until the final sample was reached.

The data recorded during enrolment include the stage and location of the DD lesion, parity, days in milk (DIM), locomotion score, breed, weight, body condition score and lameness history. Briefly, lameness was graded using a five-point scale (i.e., LS1—normal/not lame; LS2—mildly lame; LS3—moderately lame; LS4—lame; LS5—severely lame). DD lesions diagnosed clinically during examination were macroscopically classified using the modified standardised M scoring system, which comprised six levels as described by Döpfer et al. [28] and Berry et al. [29]. The classification is summarised in Table 1.

### 2.5. Allocation, Pain Assessment and Treatments Administered

No randomisation was performed in the active treatment group as all the cows received the same treatment procedure. Pain assessment was based on limb withdrawal before treatment. The cows were initially allowed to remain standing in the trim chute for 5 min. Upon immobilising the affected limb, limb withdrawal was assessed using a hoof tester by exerting mechanical pressure on the claw zones, including the skin adjacent to the DD lesion. Limb withdrawal was considered positive when the cow exhibited mild twitches or attempted to adduct the restrained limb.

**Figure 1:**
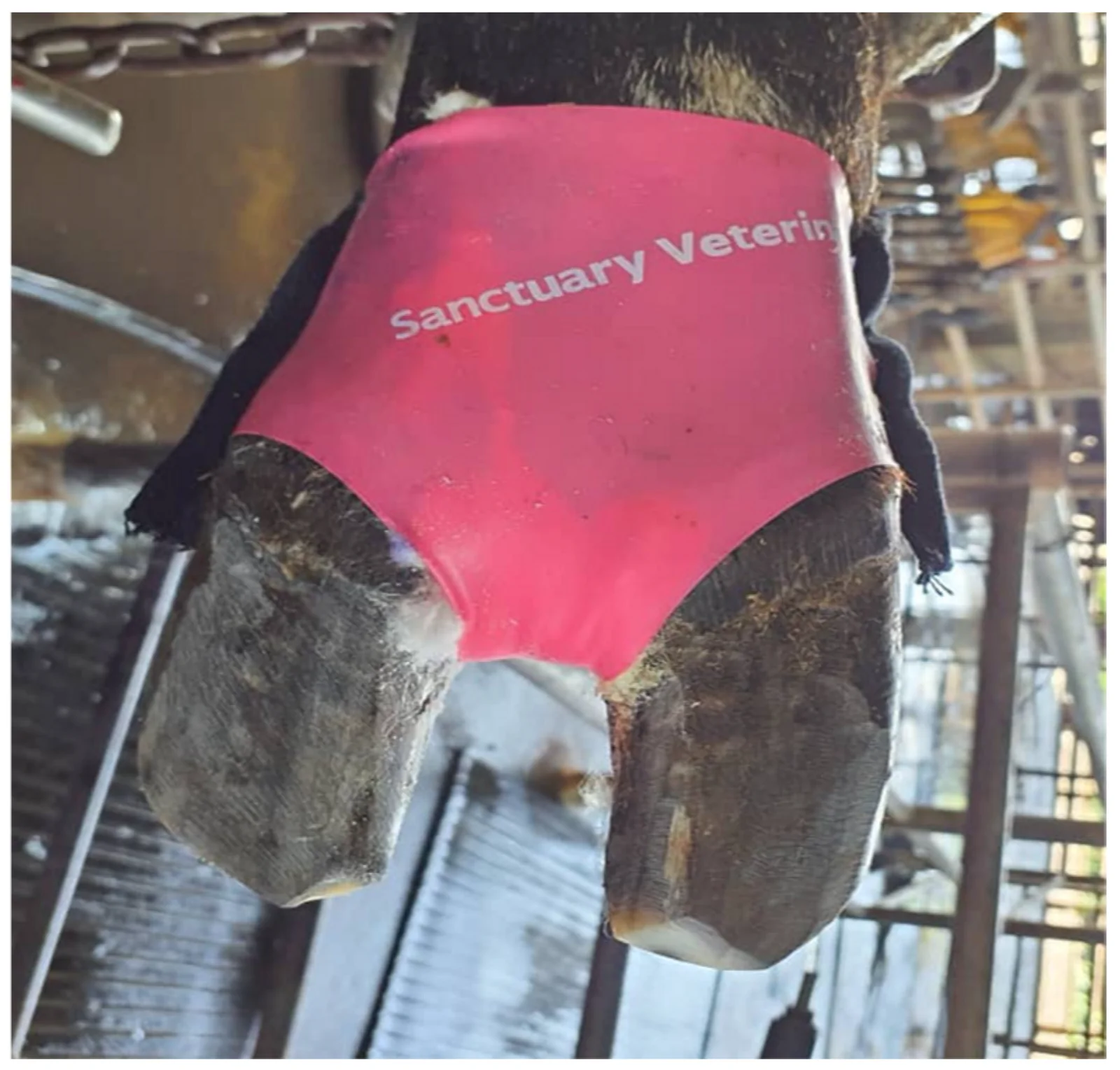
The Cow Hoof Cover**^®^** tested as a wrapping material in this study.

The standard treatment performed was according to the farm’s existing treatment modality for active DD lesions. This involved the Dutch five-step hoof-trimming method, which focused on removing diseased horn and ensuring a balance of the heel height and sole thickness for even weight distribution between the medial and lateral claws [30]. The lesion was carefully cleaned with chlorhexidine and dried with a clean paper towel, followed by debridement and removal of necrotic tissues from the lesions. Thereafter, a topical antibiotic containing oxytetracycline (Engemycin 25 mg/mL) was applied to the lesion site, followed by epidermal spray and cotton wool to wrap the lesion and the surrounding site, thereby serving as the primary or treatment layer. The final wrapping was performed using the CHC material, playing the role of a secondary and tertiary layer. Briefly, the CHC is made up of a latex, flexible material that fits directly into the interdigital space and typical site of DD lesion as shown in Figure 1. It has low permeability and is about 50 mm (2 inches) in width, water-resistant, and fixed above the coronet and mid-way between the dew claw and heel bulb when worn.

**Figure 2:**
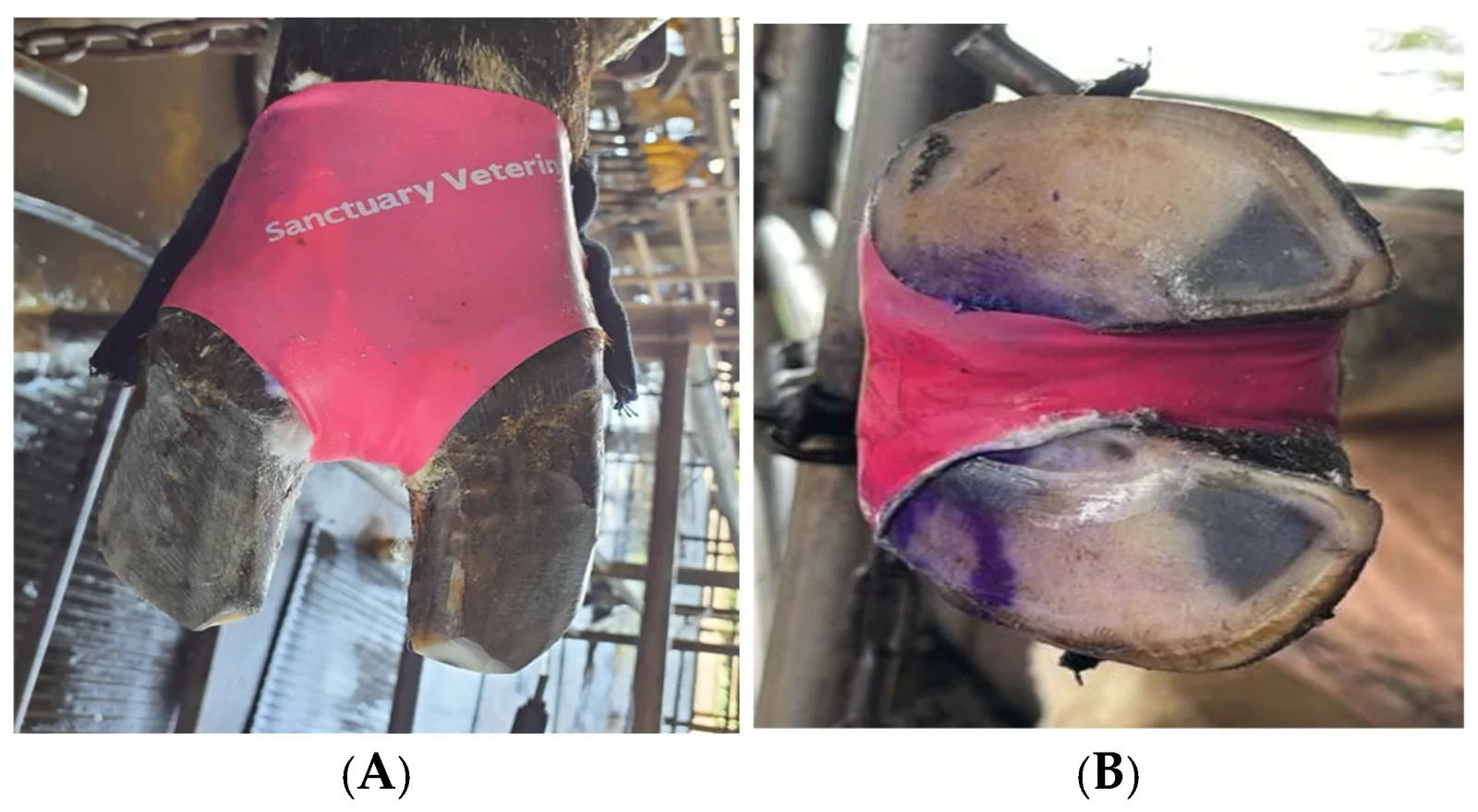

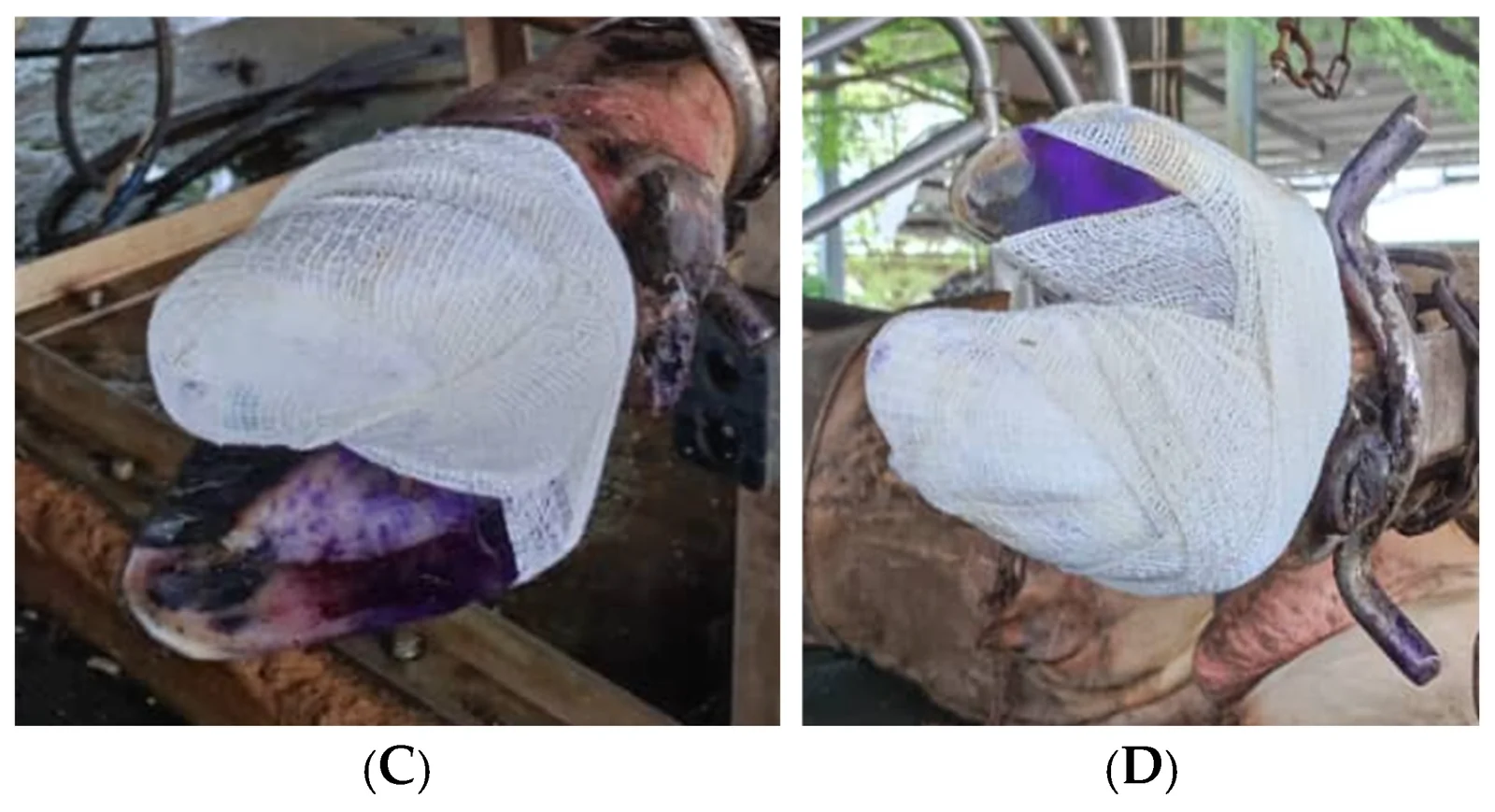
Final dressing of the DD lesions in the CHC group ((**A**): ventral view; (**B**): dorsal view; (**C**): posterior view) and conventional bandaging ((**C**,**D**) = dorsal views).

Only one wrap was applied during the first treatment. At follow-up, the wrap was removed to examine the lesion and replaced with a new one. This process was performed for all active lesions until they were completely cured. The control group received the same treatment protocol, except that a conventional bandage replaced the CHC material (Figure 2). The number of wraps and dressing materials used during the treatment and observation period was also recorded for each sample. We adhered to the farm’s standardised dressing modality, which entailed three layers comprising the primary (treatment), secondary (holding and padding) and tertiary (protective outer) layers.

All cows received an intramuscular injection of flunixin meglumine (2.2 mg/kg), administered into the neck muscles. The time spent performing the treatment was documented for each cow. Excluding the IM administration of flunixin meglumine which was performed by the farm veterinarian, all other treatment procedures for each animal in both groups were administered by two professional hoof trimmers. These hoof trimmers were trained to perform treatment for DD lesions consistently.

### 2.6. Outcome Measures and Follow-Up

The healing of claw lesions and locomotion scores at Day 28 post-intervention were the primary outcomes assessed in both groups. Progression and healing of the lesion were assessed by the same veterinarian who performed the clinical diagnosis of DD lesions. The healing of DD was based on the transition from any of the active stages to the M0 stage. As for locomotion scores, the assessor was not involved in the treatment but was responsible for recording and updating the farm data on treated lame cows. A lame cow was assumed to have recovered once a sound LS (score 1) was obtained following 2 successive observations. The follow-up periods for the two primary outcomes were at three time points after treatment, i.e., 14, 21 and 28 days after treatment. If the primary outcomes were absent day 28 post-intervention, the farm staff also documented the date on which complete healing of DD and restoration of normal gait was achieved. Thus, the total number of days being lame and the duration for complete recovery of the claw lesion were computed for each cow. Cows that were culled before the end of the study were also recorded. In the CHC group, the maintenance of CHC material and limb withdrawal was also recorded. The maintenance of CHC material refers to the presence of the CHC after treatment (i.e., CHC not detached), specifically on day 1, 3, and 7 after treatment. Limb withdrawal was used to assess changes in pain perception at week 2, 3 and 4. The CHC material or bandaging was assessed daily during the first week after treatment and it was reapplied in those without the primary dressing during this period. Cows showing a deterioration of locomotion score, or exacerbation of underlying lesions, were treated using the same treatment protocol during enrolment.

### 2.7. Data Collection and Monitoring

Data for all the animal-based characteristics and follow-up observations were recorded on a data collection sheet provided to the farm staff involved in the project. Data were then transferred to a Microsoft Excel sheet and sorted for further analysis. The researchers conducted monthly visits to the farm to monitor the research progress, which entailed the reassessment of the cow enrolment, treatment protocol, recording of outcome measures, and data collection process. Challenges faced between each visit were discussed and addressed accordingly. Video recordings and images were evaluated to ensure adherence to the treatment protocol and outcome measurement.

### 2.8. Data Analysis

Data analysis was performed using SPSS version 30. The dataset was summarised using descriptive statistics. Continuous outcome data were checked for normality using the Kolmogorov–Smirnov and Shapiro–Wilk tests before selecting the appropriate inferential statistical tests. Baseline comparisons of animal characteristics (body condition scores, BCS; days in milk, DIM) and outcome measures were conducted using either Pearson’s chi-square test or independent *t*-test for categorical and normally distributed data, respectively. Mean and standard deviation (SD) were presented for continuous variables. Subsequently, generalised estimated equations (GEE) were applied to compare the cure rates of DD and locomotion scores between the CHC and control groups at baseline and during the follow-up points (Week 1 to Week 4). We also performed survival analysis with the log-rank test to compare the transition time from active DD lesions (M1 and M2) to the M0 stage. A Cox regression model was used to assess the factors influencing the probability of DD cure. The degree of the association was measured using the hazard ratio (HR) at 95% confidence interval and a *p*-value of 0.05 was selected for a significant association between the tested variables and outcome measure. Model fit was assessed by checking overall goodness-of-fit, particularly the likelihood ratio test and the model’s predictive discrimination using Harrell’s concordance index.

## 3. Results

### 3.1. Descriptive Findings

Ten cows were enrolled between 8 October 2024 and 5 December 2024, 19 cows were enrolled between 1 January 2025 and 19 April 2025, leading to a total of 29 cows in the CHC group. Meanwhile, 27 cows were enrolled in the control groups. The CHC and control groups were similar in terms of BCS and DIM, with no significant difference (*p* > 0.05) between the groups. Likewise, parity (*p* = 0.45), locomotion score (*p* = 0.55), and location of affected limb (*p* = 0.60) were not significantly different between the CHC and control groups (Table 2). The majority of cows had M1 lesions (82.1%) during recruitment relative to M2 lesions (17.9%), but the distribution was similar in the CHC and control groups (*p* = 0.22). The mean number of bandaging/wraps used per animal was 2.4 (SD = 0.65), amounting to approximately 3 in the CHC group and 2 in the control (*p* = 0.29).

### 3.2. Effects of CHC on Locomotion Score and DD Cure

The locomotion scores decreased significantly from baseline (week 1) until week 4 in CHC and control groups (*p* < 0.001). However, changes in locomotion score were not significantly different between the groups (*p* = 0.23) during the study period (Table 3).

**Figure 3:**
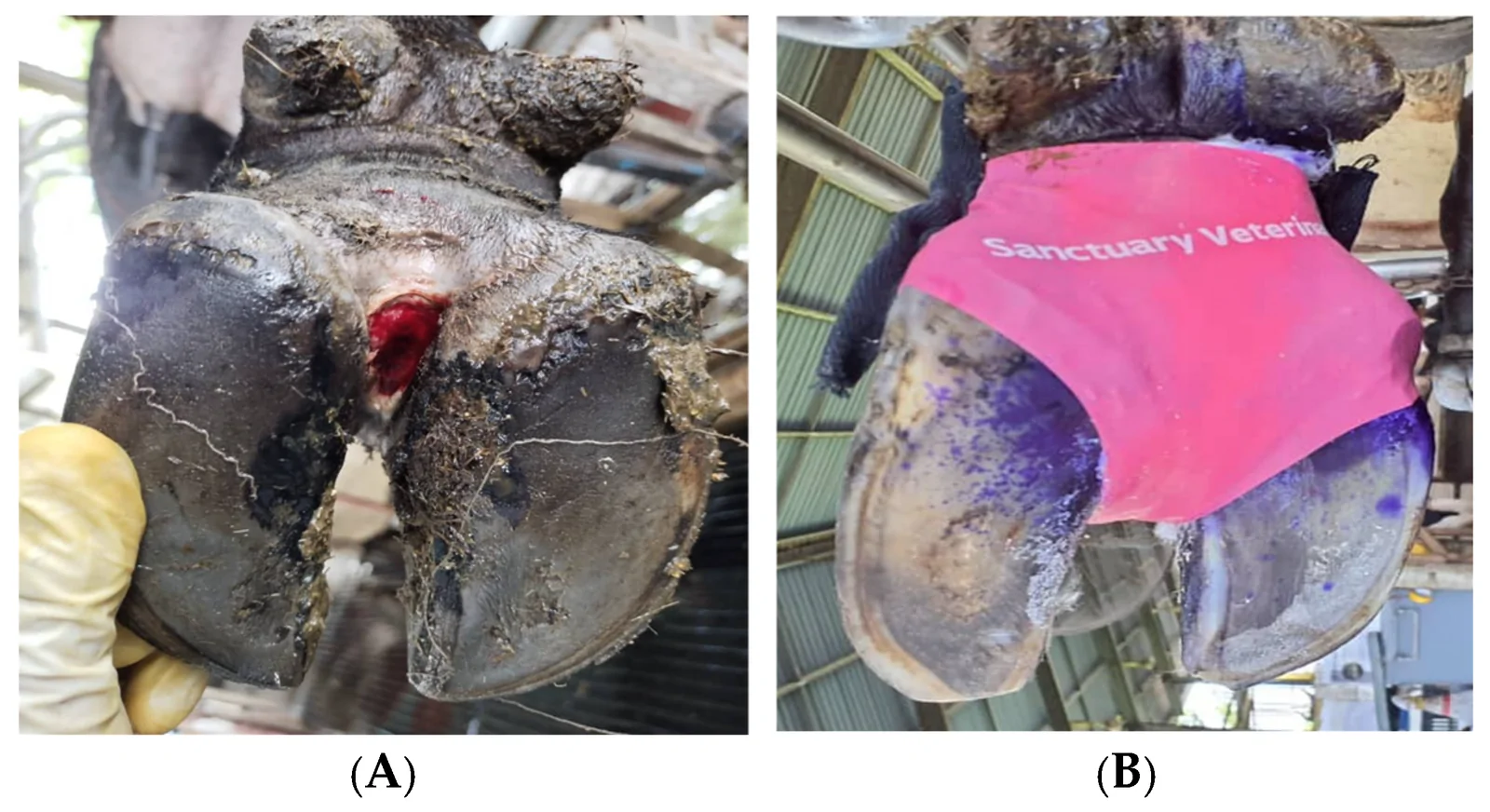

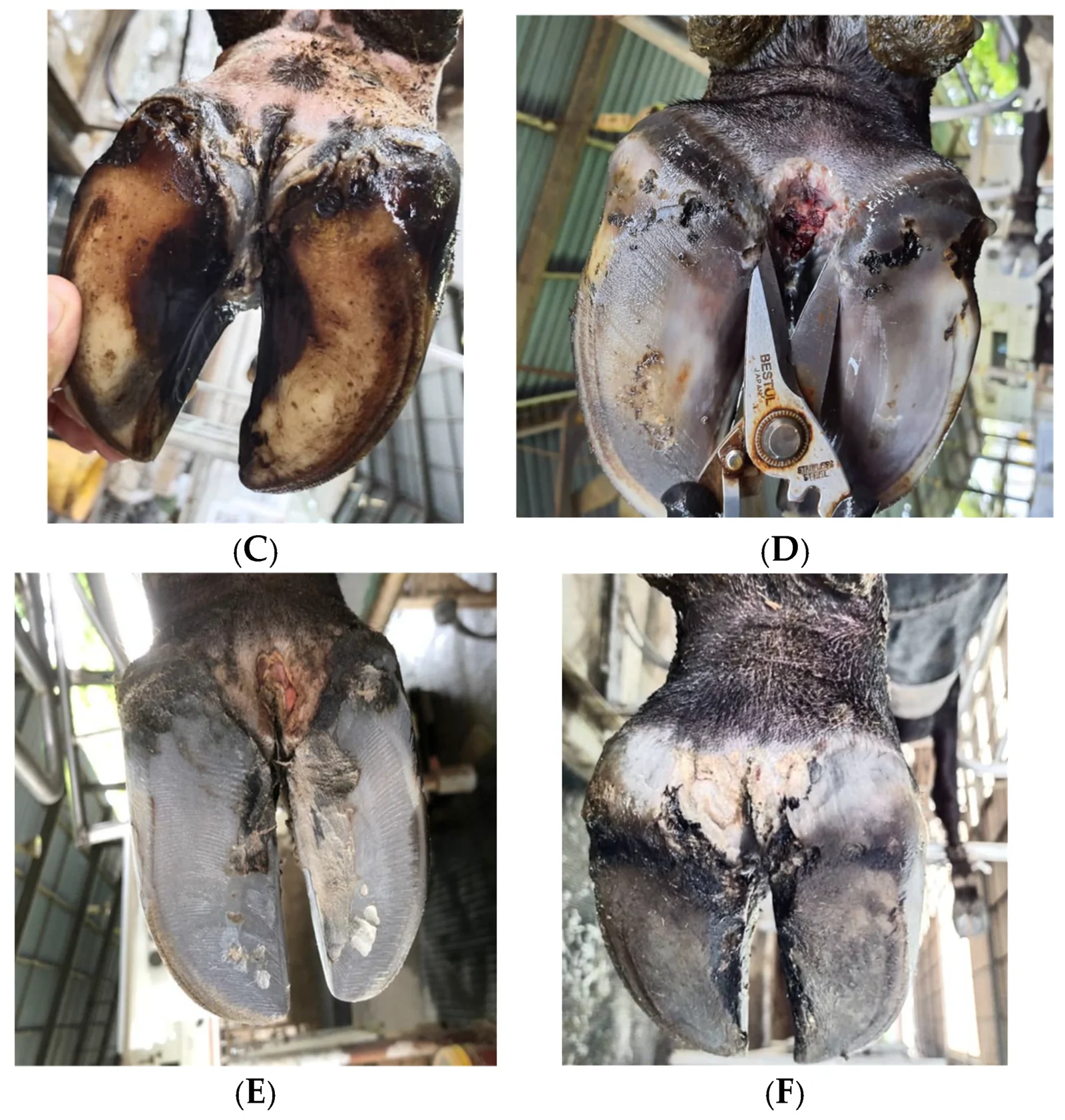
(**A**) = active M2 stage of DD lesion diagnosed during foot examination in the CHC group; (**B**) = treatment of the DD lesion and wrapping with the CHC; (**C**) = complete healing and cure (M0 stage) at Week 4 (Day 28) after treatment; (**D**) = active M2 stage of DD lesion diagnosed during foot examination in the control group; (**E**) = progression to M3 stage at Week 3 after treatment; (**F**) = progression to M4, proliferative stage, at Week 4 (Day 28) after treatment.

Table 4 presents the proportion of cows with various stages of DD lesion during the weekly observations. No significant difference was detected between the groups at baseline (week 1) (*p* = 0.12). A higher number of cows in the control group had M2 lesions (*n* = 8) at week 2, but the difference was not statistically significant (*p* = 0.06). At week 3, the CHC group had a significantly higher (*p* = 0.002) proportion of cured cows (M0; *n* = 6; 20.6%) compared to the control (*n* = 0). Likewise, the proportion of cured cows (*n* = 17; 58.6%) was significantly greater in the CHC than the control group (*n* = 7; 25.9%) at week 4. Four cows in the control group transited from M2 or M1 stage to M3 and M4 (Figure 3), but such transitions were absent in the CHC group. Overall, the CHC demonstrated 2.79 higher odds of being cured (95% CI 1.30–5.97) than the control group.

The results are further elucidated in Table 5, as the GEE analysis revealed significant main group (*p* = 0.040) and time effect (*p* = 0.001). These findings reflect that the CHC facilitated higher proportions of healed lesions than the control group across weeks, particularly at week 3 after treatment.

### 3.3. Survival and Cox Regression Analyses

**Figure 4:**
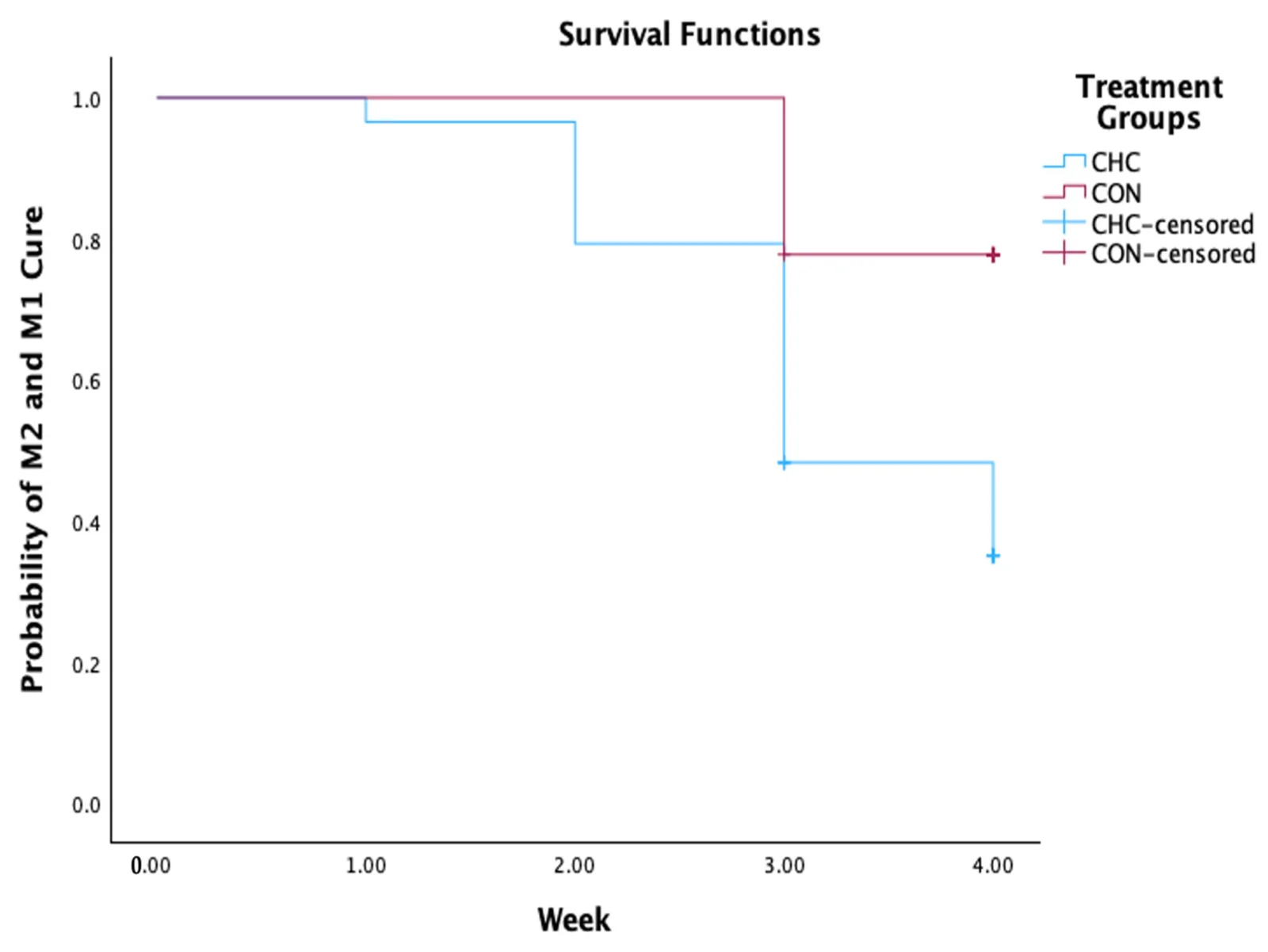
Kaplan–Meier survival analysis plots for DD lesions stratified by treatment groups and transition from active stage (M1 and M2) to cure (M0).

**Figure 5:**
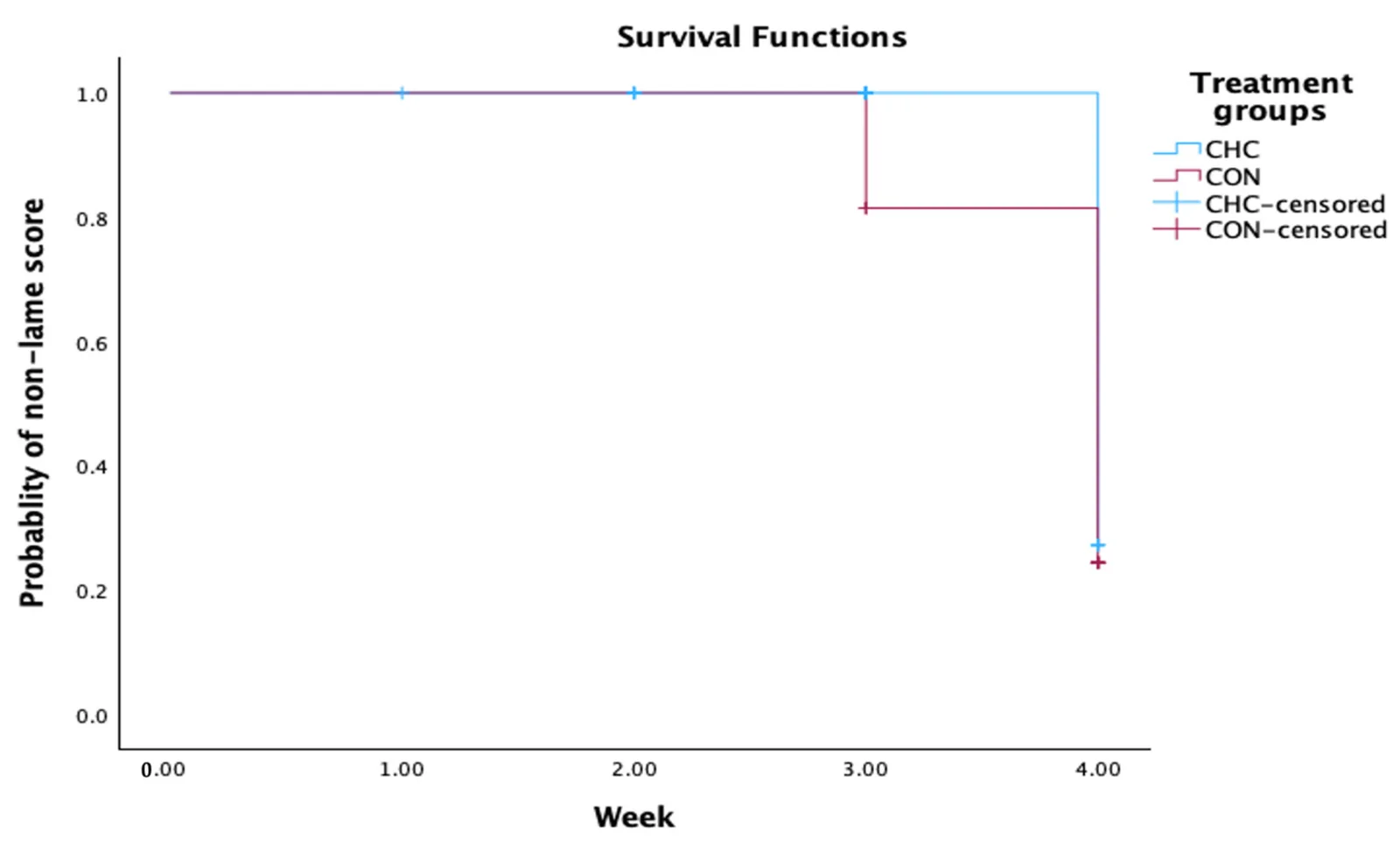
Kaplan–Meier survival analysis plots for locomotion scores stratified by treatment groups and transition from lame to non-lame scores.

The Kaplan–Meier survival curves for changes in DD cure and locomotion scores in the CHC and control groups are presented in Figure 4 and Figure 5, respectively. The control group spent approximately 4 weeks (Mean = 3.77) to record a cure from DD (M0), leading to a significantly higher mean survival time compared to the CHC (*p* = 0.001). At week 4, 59% of CHC (*n* = 17) and 26% of control (*n* = 7) were deemed macroscopically healed by week 4. The probability of cure was significantly higher in the CHC (HR = 3.57; 95% CI 1.41–9.09) relative to the control. In contrast, the survival functions and Cox-regression analysis revealed no significant effect of CHC on locomotion scores (*p* = 0.19 and *p* = 0.40) (Table 6).

Among all the variables tested in the multivariate Cox proportional regression model, only the CHC had a significant effect (HR = 3.54; 95% CI 1.05–10.52) on the likelihood of cure (transition from M2 or M1 to M0 stage). Other factors, such as parity, locomotion scores, location of affected limb, limb withdrawal reflex, and number of dressing materials used per cow had no significant effect on the probability of cure (Table 7).

## 4. Discussion

This study aimed to compare the effects of CHC and conventional bandaging as dressing materials for the treatment of active DD lesions. The hypotheses investigated were that the combination of CHC, topical treatments and flunixin meglumine will lead to higher cure rates, reduced pain scores and better restoration of cows’ gait than conventional bandaging. Research has shown that the cure of DD lesions is strongly influenced by the type of dressing [20,21,22,23]. Applying a dressing reinforced with cotton, gauze, and compresses heightens the odds of DD healing by protecting the lesion against external factors [21,31]. Before bandaging treatment, the type of dressing to be utilised, environmental factors, material costs, time and skill in removing the dressing, when necessary, must be ascertained to prevent complications and facilitate healing [19,31].

In this study, we applied a topical antibiotic containing tetracycline, which is commonly used by veterinarians and hoof trimmers for DD management. This treatment approach is often employed as a positive control in clinical trials using different application techniques with varying results. Clinical cure rates reported in treatment regimens involving tetracycline are around 60% over 3 to 30 days [32]. Cutler et al. [23] reported that a tetracycline hydrochloride paste had therapeutic effects that were comparable to the combination of tetracycline hydrochloride and bandage for two days, thus eliminating the need for bandage removal.

Both groups showed improvement in gait across time and the transition from lame score to non-lame score was not significantly different between the groups. However, the DD cure rate was significantly higher in the CHC group compared to the control, particularly at Week 3 and Week 4 post-intervention. The finding suggests that healing of DD lesions occurred faster in the CHC group, with an overall greater cure rate compared to bandaged lesions, regardless of the antibiotic and NSAID treatments administered. Notably, active DD lesions in approximately 59.0% of cows in the CHC group were completely healed (cure rate of painful ulcerative M2 lesion) at week 4 relative to 26.0% in the bandage group. Moreover, only the control group had cows that transited from M2 or M1 stage to M3 and M4. Thus, while the proportion of completely healed cows was similar in both groups during the first and second week, the CHC group experienced more rapid healing during the third week after treatment. The possible explanation is that CHC was effective in reducing pain from ulcerative lesions and promoting healing, evidenced by the restoration of normal gait.

The overall cure rate at week 4 is relatively lower than that reported in a previous study [21], whereby 71% to 86% of lesions were completely cured in bandaged antibiotic and intra-hoof gel treatment groups. The researchers used a more complex bandaging technique, involving the addition of a layer of beech tar to the padded bandage. The dressing might have stabilised and prevented the bandage from absorbing moisture, as well as protecting the lesion and facilitating prolonged exposure to treatment. The non-bandaged group in the study by Klawitter et al. [21] recorded only a 30% cure rate within the same period. Meanwhile, Mudroň et al. [33] found no significant difference in the treatment success between non-bandaged and bandaged techniques, as all groups recorded a 100% success rate. Likewise, Mendonça et al. [34] revealed that bandaging of DD lesions had no beneficial effect on locomotion scores compared to spraying alone or the combination of spray and bandage in the first 3 days. Notably, crepe bandages were not effective in protecting the injuries from environmental humidity, as the dressings were damped during follow-up examinations. Differences in the study design, definition of cure rates, environmental factors (i.e., barn cleanliness and moisture), and severity and anatomical location of DD lesions in enrolled cows may explain some of these discrepancies. For instance, the definition of cure rate by Mudron et al. [33] was the transition of DD lesions from active to non-active stages, and all the enrolled cows had mild lesions with no significant disturbance in the locomotion scores. Cure rates and lesion progression were not assessed by Mendonça et al. [34] because the last follow-up was on the 10th day, which is insufficient for complete cure. Nevertheless, all the studies reflect the significance of bandaging DD lesions combined with topical antibiotic agents [21,33].

The fact that most cured DD lesions in our study occurred in the CHC group within the first three weeks suggests that longer treatment may be less effective. This event was more pronounced in the bandage group, as the majority of cows spent at least a month before the lesion was completely cured. More importantly, higher proportions of cured lesions at week 3 in the CHC group highlight the potential effect of the dressing material in reducing the transition of M2 into M4 lesions. Bandaging also demonstrated a similar effect, but at a cost of a prolonged duration compared to the CHC group. Klawitter et al. [21] also reported the significant effect of bandaging on the progression of M2 to M4 lesions. This effect is widely believed as key in DD control, given that M4 lesions are more likely to be hyperkeratotic relative to M2 lesions [29,34,35], thereby complicating the penetration and therapeutic effects of topical agents [36]. Moreover, recurrent lesions are common in cows with a history of M4 lesions, which are often presented as open lesions on proliferated skin (M4.1) [29].

The bandaging technique and the application of CHC used in this study are practical for farmers, unlike the bandaging process employed in prior studies [21]. Comparatively, conventional bandaging requires skills during application and removal applying to either protect or prevent ischaemic injuries [37]. The CHC, on the other hand, is easy to apply and stays for at least 48 h, which is considered adequate for protecting the lesion and ensuring optimal exposure to topical agents. Thus, the CHC is more practical in terms of time and application, offering important information to farmers for informed decision-making on treatment cost and resources.

### Strengths and Limitations

This study presents an opportunity to ascertain the efficacy of a novel dressing material for DD management in dairy cows. A quasi-experimental design was applied, including the use of standardised diagnostic and treatment approaches for the intervention and control groups, and attempts to establish equivalence in both groups. Enrolled animals were also followed closely for lesion progression and treatment outcomes, including nociception and gait restoration. However, it is crucial to consider the research limitations for cautious interpretation of the findings.

Baseline equivalence was achieved between the groups based on the severity of DD cases and animal-based characteristics. This was strengthened by randomly selecting historical controls of DD cases diagnosed and treated by the same personnel within 3 months prior to the clinical trial and under the same environmental and management conditions. Nevertheless, a prospective randomised control trial (RCT) in which newly diagnosed DD cases are allocated concurrently to either CHC or conventional bandaging will completely eliminate potential selection bias and establish comparability between the groups.

Transition monitoring of DD cases is vital in the disease management and establishing complete cure. In a clinical trial conducted by Alsaaod et al. [38], the treatment of DD lesions with a combination of salicylic acid paste and bandages led to the regression of stage M2 lesions to M0 within four weeks and PCR results confirmed the elimination of *Treponema* from deep epidermal layers. Our treatment in the control group consists of therapeutic trimming, topical antibiotics, NSAID and bandaging, which is similar to the treatment approach used by Alsaaod et al. [38]. The rapid regression from active to non-active stages in the CHC group suggests the promising role of the dressing in addressing animal-based and environmental factors that might affect the healing rate. In this study, the effect of the treatment on susceptible and resistant stages could not be elucidated because active lesions were grouped together (M1, M2 and M4.1). Future studies may focus on investigating the effect of various treatments on specific stages of DD.

Recurrence of DD is another important event that was not investigated in this study, given the short-term assessment, as recurrence of DD takes a longer period (>10 weeks) [39]. Although the treatment applied may facilitate lesion healing, the likelihood of new DD infections following skin irritation cannot be ruled out. Our study measured clinical cure rates without examining the presence or absence of treponemes or other associated bacteria. The detection of the presence of treponemes in histological samples and microbiological analyses was beyond the present study scope. Despite results suggesting that the transition of M2 lesions into chronic stages may be prevented by different bandaging materials, the potential persistence of treponemes on the skin may trigger recurrent infections. Berry et al. [29] reported that all DD lesions receiving bandaged lincomycin treatment healed histologically by day 12, but approximately half were positive for treponemes by days 23 and 37, confirming cases of recurring infections. Future research should investigate histological long-term reoccurrence rates of CHC bandaged DD lesions.

To assess pain response in the treated cows, a hoof-testing technique was performed in this study by applying mechanical pressure on the sensitive areas of the affected claw. This method is similar to pressure algometry used to quantify pain during calf disbudding [40], lame sheep [41] and cattle affected with sole ulcers and white line disease [42]. No significant pain response was observed in cows with healed lesions, whereas a lower nociceptive threshold was detected in active lesions (during enrolment and early follow-up periods), indicating ongoing pain. However, different stages of disease progression may cause varying pain intensity. Future research using the lesion severity scale and objective pain assessment methods, like quantification of pain biomarkers, could provide more insight into pain during DD infection. More frequent examination might also provide further details about healing.

No control or a placebo group was recruited in this study for animal welfare reasons. M2 lesions are usually the most painful stage of DD, and treatment with water spray or bandaging alone after cleaning with a dry towel as a type of placebo has no effect in promoting healing [31]. To minimise animal distress, we compared the effects of two types of dressing materials and treatment with NSAIDs and topical antibiotics. Nonetheless, we acknowledge the importance of having a control group since various farm-level and management factors may influence the cure rates. Additional treatment groups could be established by cleaning the lesion, drying with a towel, and applying either CHC or conventional bandaging without topical antibiotics.

## 5. Conclusions

This field experiment depicts the potential of a novel dressing material, CHC, as a treatment modality for DD in dairy cattle, evidenced by the rapid healing of lesions between days 21 and day 28 post-application. The promising role in facilitating recovery from DD-associated lameness was evident, as treated cows experienced lower locomotion scores at day 14 until day 28 compared to those managed using conventional bandaging. The CHC remained intact at least until day 3 post-application, and the positive effects on DD lesions are bolstered when it is changed after this period. However, further studies involving histological investigation and robust clinical trials are recommended to understand its efficacy for complete cure.

## Figures and Tables

**Table 1:** A simplified classification and description of various stages of DD applied in this study.

Stage	Simplified Classification	Main Appearance/Signs
M0	Normal/no lesion	Healthy skin; no visible signs of digital dermatitis.
M1	Early/small lesion	Small, usually superficial lesion (typically <2 cm); may be red and moist.
M2	Active lesion	Large, active, painful lesion; often red, ulcerated, moist, and may have a “strawberry-like” appearance.
M3	Healing lesion	Previously active M2 lesion is healing; covered by a scab or firm tissue, with reduced inflammation.
M4	Chronic lesion	Chronic changes such as thickened, raised, hairless or wart-like skin; may persist for a long time.
M4.1	Chronic lesion with new active lesion	An M4 chronic lesion with a new small active M1 lesion developing within or beside it.

**Table 2:** Characteristics of the cows in the CHC and Control groups.

	CHC (*n* = 29)	Control (*n* = 27)	Overall (*n* = 56)	*p*-Value
DIM (days) (Mean ± SD)	268.13 ± 178.22	179.70 ± 180.15	225.50 ± 183.03	0.70 ^a^
BCS (average score)	3.54 ± 0.23	3.51 ± 0.11	3.53 ± 0.18	0.72 ^a^
Treatment duration, minutes (Mean ± SD)	23.7 ± 6.36	24.2 ± 7.29	24.00 ± 6.76	0.78 ^a^
Parity				
Heifers (%)	6 (20.7)	8 (29.6)	14 (25.0)	0.45 ^b^
Primiparous (%)	10 (34.5)	8 (29.6)	18 (32.1)	
Second parity (%)	8 (27.6)	10 (37.0)	18 (32.1)	
>Second parity (%)	5 (17.2)	1 (37.0)	6 (10.7)	
Locomotion score				
Not lame (LS1) (%)	0 (0.0)	6 (22.2)	6 (10.7)	0.55 ^b^
Mildly lame (LS2) (%)	15 (51.7)	14 (51.8)	29 (51.7)	
Lame (LS3) (%)	14 (48.3)	7 (25.9)	21 (37.5)	
Location				
Hindlimb (%)	25 (86.2)	20 (74.0)	45 (80.4)	0.60 ^b^
Forelimb (%)	4 (13.8)	7 (26.0)	11 (19.6)	
M stage of DD				
M1	26 (89.6)	20 (74.1)	46 (82.1)	0.22 ^b^
M2	3 (10.4)	7 (25.9)	10 (17.9)	
Number of bandaging/dressing material used per animal (Mean ± SD)	2.52 (0.78)	2.33 (0.48)	2.42 ± 0.65	0.29 ^a^

Notes: BCS—body condition scores; CHC—Cow Hoof Cover**^®^**; DIM—days in milk; LS—locomotion score; ^a^—by independent *t*-test; ^b^—by Pearson’s chi-square test; SD-standard deviation.

**Table 3:** Comparison of locomotion scores between the CHC and control groups.

Parameter		B	SE	95% Wald Confidence Interval		Hypothesis Test		
				Lower	Upper	Wald Chi-Square	df	*p*-Value
Locomotion scores	LS1	−1.39	0.41	−2.20	−0.59	11.59	1	<0.001
	LS2	0.62	0.35	−0.07	1.30	3.11	1	0.078
	LS3	2.83	0.67	1.51	4.15	17.72	1	<0.001
CHC		−0.48	0.39	−1.26	0.29	1.46	1	0.23
Control		Reference						

Notes: B—coefficient; CHC—Cow Hoof Cover**^®^**; df—degree of freedom; LS—locomotion score; LS1—sound; LS2—mild lameness; LS3—moderate lameness; SE—standard error.

**Table 4:** Distribution of cows with active DD and cured lesions across time points (Week 1 to Week 4) in the CHC and control groups.

Time/Groups	M1	M2	M3	M4	M4.1	M0	Chi-Square	*p*-Value	OR (95% CI)
Week 1 (Baseline)									
CHC (%)	26 (89.6) ^a^	3 (10.3) ^a^	0	0	0	0	2.31	0.12	0.39 (0.11–1.38)
Control (%)	20 (74.0) ^a^	7 (25.9) ^a^	0	0	0	0			
Week 2									
CHC (%)	26 (89.6) ^a^	2 (6.9) ^a^	0	0	0	1 (3.4) ^a^	5.63	0.06	
Control (%)	19 (70.3) ^a^	8 (29.6) ^a^	0	0	0	0 ^a^			
Week 3									
CHC (%)	22 (75.8) ^a^	1 (3.4) ^a^	0	0	0	6 (20.6) ^a^	12.74	0.002	
Control (%)	18(66.7) ^a^	9 (33.3) ^b^	0	0	0	0 ^b^			
Week 4									
CHC (%)	10 (34.4)	2 (6.9)	0	0	0	17 (58.6)	8.69	0.04	
Control (%)	13 (48.1)	3 (11.1)	2 (7.40)	2 (7.40)	0	7 (25.9)			
Groups	Cured	Not cured							
CHC (%)	18 (62.0)	11 (38.0)	0	0	0		9.06	0.003	2.79 (1.30–5.97)
Control (%)	6 (22.2)	21 (77.7)	0	0	0				

Notes: CI—confidence interval; CHC—Cow Hoof Cover**^®^**; M0–M4.1—stages of digital dermatitis lesions; OR—odds ratio, ^a^ and ^b^ are superscripts comparing the frequency or proportion of specific DD stage between CHC and control, with different superscripts indicating significant differences between groups at each observation point. DD Stages with zero observations were not included in the chi-square tests.

**Table 5:** GEE analysis of the proportion of cows with cured DD cases (M0) between the CHC and control groups.

Parameter	B	SE	95% CI		Hypothesis Test	
			Lower	Upper	Wald Chi-Square	*p*-Value
Intercept	0.86	0.42	0.04	1.69	4.21	0.040
CHC	−1.21	0.56	−2.32	−0.10	4.60	0.030
Control	Referent					
Day 1	27.70					<0.001 *
Week 2	29.21	1.02	27.19	31.22	808.08	<0.001 *
Week 3	23.64	0.45	22.75	24.52	2740.76	<0.001 *
Week 4	Referent					
CHC * Day1	1.213					0.10
CHC * Week 2	−25.53	1.05	26.24	33.24	872.03	0.09
CHC * Week 3	23.64	0.34	24.57	28.42	2653.66	0.032 *
CHC * Week 4	Referent					

Notes: B = coefficient; SE = standard error; CHC = Cow Hoof Cover**^®^**; CI = confidence interval; DD = digital dermatitis; *—significant at *p*-value 0.05; CHC = Cow Hoof Cover**^®^**.

**Table 6:** Hazard ratio of M2 and M1 cure and non-lame score in the CHC and control groups.

Variables	B	SE	Wald	HR	(95% CI)	*p*-Value
DD cure						
CHC	−1.27	0.47	7.22	3.57	1.41–9.09	0.007
Control	-	-			Reference	
Non-lame score						
CHC	0.35	0.42	0.70	1.42	0.62–3.26	0.40
Control					Reference	

Notes: CHC—Cow Hoof Cover**^®^**; SE—standard error.

**Table 7:** Cox regression model for the factors associated with DD cure in the study population.

Variables	B	SE	Wald	*p*-Value	HR	95.0% CI for Exp(B)	
						Lower	Upper
Group							
CHC	−1.26	0.55	5.15	0.02	3.54	1.05	10.52
Control							
DIM	0.00	0.002	0.10	0.75	1.000	0.99	1.00
Parity			0.15	0.98			
Heifer	0.11	0.74	0.02	0.87	1.125	0.26	4.85
Primiparous	0.12	0.66	0.03	0.84	1.135	0.30	4.20
Second parity	−0.13	0.85	0.02	0.87	0.874	0.16	4.64
>Second parity	1.32	1.32	1.00	0.31	3.757	0.28	50.24
LS			1.18	0.55			
Non-lame	0.87	1.20	0.53	0.46	2.408	0.22	25.44
Mildly lame	0.38	1.29	0.08	0.76	1.463	0.11	18.63
Limb withdrawal reflex							
Present	−0.17	0.52	0.11	0.73	0.838	0.29	2.35
Absent							
Treatment duration	−0.04	0.04	0.86	0.35	0.957	0.87	1.04
Number of dressing material used	0.41	0.35	1.36	0.24	1.508	0.75	3.00

## Data Availability

The original contributions presented in this study are included in the article. Further inquiries can be directed to the corresponding authors.

## References

[1] Palmer M., O’Connell N. (2015). Digital Dermatitis in Dairy Cows: A Review of Risk Factors and Potential Sources of Between-Animal Variation in Susceptibility. Animals.

[2] Solano L., Barkema H.W., Jacobs C., Orsel K. (2017). Validation of the M-Stage Scoring System for Digital Dermatitis on Dairy Cows in the Milking Parlor. J. Dairy Sci..

[3] Sullivan L.E., Evans N.J., Blowey R.W., Grove-White D.H., Clegg S.R., Duncan J.S., Carter S.D. (2015). A Molecular Epidemiology of Treponemes in Beef Cattle Digital Dermatitis Lesions and Comparative Analyses with Sheep Contagious Ovine Digital Dermatitis and Dairy Cattle Digital Dermatitis Lesions. Vet. Microbiol..

[4] Rashid F.D.B., Mohd Roslan S.N.B., Lit Kai J.T., Ahmad Tajuddin A.B., Ramanoon S.Z., Othman A.H., Sadiq M.B. (2025). Lameness and Hoof Disorders in Sheep and Goats from Small Ruminant Farms in Selangor, Malaysia. Animals.

[5] Zinicola M., Lima F., Lima S., Machado V., Gomez M., Döpfer D. (2015). Altered Microbiomes in Bovine Digital Dermatitis Lesions, and the Gut as a Pathogen Reservoir. PLoS ONE.

[6] Kasiora K., Anagnostopoulos A., Bedford C., Menka T., Barden M., Griffiths B.E., Achard D., Timms K., Machado V.S., Coates A. (2022). Evaluation of the use of ketoprofen for the treatment of digital dermatitis in dairy cattle: A randomised, positive-controlled, clinical trial. Vet. Rec..

[7] Robcis R., Ferchiou A., Berrada M., Ndiaye Y., Herman N., Lhermie G., Raboisson D. (2023). Cost of lameness in dairy herds: An integrated bioeconomic modeling approach. J. Dairy Sci..

[8] Gomez A., Cook N.B., Socha M.T., Döpfer D. (2015). First-lactation performance in cows affected by digital dermatitis during the rearing period. J. Dairy Sci..

[9] Dolecheck K.A., Dwyer R.M., Overton M.W., Bewley J.M. (2018). A survey of United States dairy hoof care professionals on costs associated with treatment of foot disorders. J. Dairy Sci..

[10] Bell N.J., Randall L. (2021). Lameness in Dairy Heifers: A Narrative Review of Control Strategies. Livestock.

[11] De Jong E., Frankena K., Orsel K. (2021). Risk Factors for Digital Dermatitis in Free-stall-housed, Canadian Dairy Cattle. Vet. Rec. Open.

[12] Kofler J., Suntinger M., Mayerhofer M., Linke K., Maurer L., Hund A., Fiedler A., Duda J., Egger-Danner C. (2022). Benchmarking Based on Regularly Recorded Claw Health Data of Austrian Dairy Cattle for Implementation in the Cattle Data Network (RDV). Animals.

[13] Thomas A.D., Orsel K., Cortés J.A., Pajor E.A. (2022). Objective Determination and Quantification of Pain and Inflammation Associated with Digital Dermatitis in Feedlot Cattle. Appl. Anim. Behav. Sci..

[14] Laschinger J., Furtner A.-L., Fuerst-Waltl B., Pesenhofer R., Kofler J. (2025). Associations Between Locomotion Scores and Specific Claw Lesions in Dairy Cows from Digital Dermatitis-Infected Herds. Animals.

[15] Bergsten C., Blowey R.W., Capion N., Fiedler A., Geldhof J., Holzhauer M., Kloostermann P. Relun A: Five-Point Plan for Control of Digital Dermatitis. Proceedings of the British Cattle Lameness Conference.

[16] Weber J., Hillen M., Seuberlich T., Fürmann A., Gurtner C., Becker J., Syring C., Ruiters M.W., Alsaaod M., Mazurek L. (2025). Successful implementation of a risk assessment and mitigation program to control bovine digital dermatitis at the herd-level. Sci. Rep..

[17] Bell N.J., Main D.C.J. (2012). Case report: Reducing lameness through active screening, attention to details and skilled staff. Livestock.

[18] Evans N.J., Murray R.D., Carter S.D. (2016). Bovine digital dermatitis: Current concepts from laboratory to farm. Vet. J..

[19] Potterton S.L., Bell N.J., Whay H.R., Berry E.A., Atkinson O.C.D., Dean R.S. (2012). A descriptive review of the peer and non-peer reviewed literature on the treatment and prevention of foot lameness in cattle published between 2000 and 2011. Vet. J..

[20] Hayton R., Britten N., McCrone I. (2025). A randomised controlled field trial of an adhesive gel bandage in addition to topical antibiotic treatment of digital dermatitis in dairy cows. AgriRxiv.

[21] Klawitter M., Braden T.B., Müller K.E. (2019). Randomised clinical trial evaluating the effect of bandaging on the healing of sole ulcers in dairy cattle. Vet. Anim. Sci..

[22] Dotinga A., Jorritsma R., Nielen M. (2017). A randomised non-inferiority trial on the effect of an antibiotic or non-antibiotic topical treatment protocol for digital dermatitis in dairy cattle. Vet. Evid..

[23] Cutler J.H., Cramer G., Walter J.J., Millman S.T., Kelton D.F. (2013). Randomised clinical trial of tetracycline hydrochloride bandage and paste treatments for resolution of lesions and pain associated with digital dermatitis in dairy cattle. J. Dairy Sci..

[24] Fiedler A., Sauter-Louis C., Maierl J. (2015). Polyurethane dressing, tetracycline and salicylic acid used for the treatment of digital dermatitis in cattle. A comparative study. Tierärztl. Prax. Ausg. G Grosstiere Nutztiere.

[25] Chiba K., Miyazaki T., Sekiyama Y. (2017). The therapeutic efficacy of allyl isothiocyanate in cows with bovine digital dermatitis. J. Vet. Med. Sci..

[26] Sprecher D.J., Hostetler D.E., Kaneene J.B. (1997). A lameness scoring system that uses posture and gait to predict dairy cattle reproductive performance. Theriogenology.

[27] Egger-Danner C., Nielsen P., Fiedler A., Müller K., Fjeldaas T., Döpfer D., Daniel V., Bergsten C., Cramer G., Christen A.M. (2015). ICAR Claw Health Atlas. ICAR Tech. Ser..

[28] Döpfer D., Koopmans A., Meijer F.A., Szakáll I., Schukken Y.H., Klee W., Bosma R.B., Cornelisse J.L., van Asten A.J., Ter Huurne A.A. (1997). Histological and bacteriological evaluation of digital dermatitis in cattle, with special reference to spirochaetes and Campylobacter faecalis. Vet. Rec..

[29] Berry S.L., Read D.H., Famula T.R., Mongini A., Döpfer D. (2012). Long-term observations on the dynamics of bovine digital dermatitis lesions on a California dairy after topical treatment with lincomycin HCl. Vet. J..

[30] Sadiq M.B., Ramanoon S.Z., Shaik Mossadeq W.M., Mansor R., Syed-Hussain S.S. (2021). A modified functional hoof trimming technique reduces the risk of lameness and hoof lesion prevalence in housed dairy cattle. Prev. Vet. Med..

[31] Toholj B., Stevancevic M., Kos J. (2012). Efficiency of different therapeutic protocols in treating digital dermatitis in dairy cows. Vet. Arch..

[32] Logue D.N., Gibert T., Parkin T., Thomson S., Taylor D.J. (2012). A field evaluation of a footbathing solution for the control of digital dermatitis in cattle. Vet. J..

[33] Mudroň P., Coles L.L., Réková P. (2021). Use of bandaging in the treatment of digital dermatitis. Folia Vet..

[34] Mendonça A.P.A., Teodoro P.H.M., Silva J.R.B., Moura R.B.R., Ravetti R., Rodrigues C.A. (2024). Effectiveness of HoofCare^®^ in the treatment of digital dermatitis in dairy cows. Arq. Bras. Med. Veterinária Zootec..

[35] Klitgaard K., Boye M., Capion N. (2008). Evidence of multiple Treponema phylotypes involved in bovine digital dermatitis as shown by 16S rRNA gene analysis and fluorescence in situ hybridisation. J. Clin. Microbiol..

[36] Schultz N., Capion N. (2013). Efficacy of salicylic acid in the treatment of digital dermatitis in dairy cattle. Vet. J..

[37] Anderson D.M., White R.A. (2000). Ischemic bandage injuries: A case series and review of the literature. Vetinary Surg..

[38] Alsaaod M., Jensen T.K., Miglinci L., Gurtner C., Brandt S., Pluss J. (2022). Proof of an optimised salicylic acid paste-based treatment concept of ulcerative M2-stage digital dermatitis lesions in 21 dairy cows. PLoS ONE.

[39] Nielsen B.H., Thomsen P.T., Green L.E., Kaler J. (2012). A study of the dynamics of digital dermatitis in 742 lactating dairy cows. Prev. Vet. Med..

[40] Heinrich A., Duffield T.F., Lissemore K.D., Millman M.T. (2010). The effect of meloxicam on behaviour and pain sensitivity of dairy calves following cautery dehorning with a local anaesthetic. J. Dairy Sci..

[41] Ley S.J., Waterman A.E., Livingston A. (1995). A field study of the effect of lameness on mechanical nociceptive thresholds in sheep. Vet. Rec..

[42] Sadiq M.B., Ramanoon S.Z., Shaik Mossadeq W.M., Mansor R., Syed-Hussain S.S. (2022). Treatment protocols for claw horn lesions and their impact on lameness recovery, pain sensitivity, and lesion severity in moderately lame primiparous dairy cows. Front. Vet. Sci..

